# Assembly and regulation of γ-tubulin complexes

**DOI:** 10.1098/rsob.170266

**Published:** 2018-03-07

**Authors:** Dorian Farache, Laurent Emorine, Laurence Haren, Andreas Merdes

**Affiliations:** Centre de Biologie Intégrative, Centre de Biologie du Développement, CNRS-Université Toulouse III, 31062 Toulouse, France

**Keywords:** gamma-tubulin, gamma-tubulin complex proteins, CM1 proteins, microtubule nucleation, microtubule-organizing centres, centrosome

## Abstract

Microtubules are major constituents of the cytoskeleton in all eukaryotic cells. They are essential for chromosome segregation during cell division, for directional intracellular transport and for building specialized cellular structures such as cilia or flagella. Their assembly has to be controlled spatially and temporally. For this, the cell uses multiprotein complexes containing γ-tubulin. γ-Tubulin has been found in two different types of complexes, γ-tubulin small complexes and γ-tubulin ring complexes. Binding to adaptors and activator proteins transforms these complexes into structural templates that drive the nucleation of new microtubules in a highly controlled manner. This review discusses recent advances on the mechanisms of assembly, recruitment and activation of γ-tubulin complexes at microtubule-organizing centres.

## 1. Introduction

Microtubules are tubular polymers that assemble from heterodimers of α and β-tubulin. Their formation occurs spontaneously *in vitro* from purified protein in the presence of GTP, if the concentration of *α*/β-tubulin exceeds a critical concentration. In practice, microtubules can be assembled reproducibly *in vitro*, at tubulin concentrations of approximately 20 µM [1]. This value matches well the concentration of tubulin that was measured in cytoplasmic extracts [2], raising the question why microtubules grow off specific organizing centres in the cell, instead of polymerizing ubiquitously in an uncontrolled manner. An answer may be that the formation of microtubules from pure tubulin is kinetically disfavoured. *In vitro*, microtubule assembly occurs in multiple steps: initially, a small number of tubulin dimers need to oligomerize, to form a stable nucleus with correct geometry. This is considered to be a slow process, because a dynamic equilibrium between dimers and oligomers exists, and detachment of dimers at this stage leads to immediate loss of the nucleus. However, any nucleus that has grown sufficiently large permits the longitudinal addition of new dimers, leading to rapid elongation. In cells, multiprotein complexes of γ-tubulin are used as templates for the longitudinal association with *α*/β-tubulin dimers, thus reducing the duration of the nucleation process [3]. These complexes are essential to permit the rapid formation of spindle microtubules at early stages of mitosis. Their absence leads to severe defects in spindle formation, cell cycle arrest and cell death [4–6]. As these γ-tubulin complexes are only active upon recruitment to specific microtubule-organizing centres (MTOCs) such as the centrosome, the cell possesses spatial and temporal control over the growth of microtubules.

## 2. Composition of γ-tubulin complexes

The major constituents of γ-tubulin complexes comprise γ-tubulin, a member of the tubulin family, and ‘γ-tubulin complex proteins' (GCPs). γ-Tubulin was originally discovered in the fungus *Aspergillus nidulans*, as a suppressor of a temperature-sensitive β-tubulin mutation [7]. Highly conserved homologues of γ-tubulin were identified soon afterwards in a variety of organisms [8,9], and it became clear that γ-tubulin would be a universal component involved in the nucleation and organization of microtubules. Two genes encoding γ-tubulin isoforms were identified in *Drosophila* and in vertebrates [6,10]. In mice, only one isoform, TUBG1, was found essential and ubiquitously expressed in the body [6]. GCPs were first identified in biochemical purifications of γ-tubulin-containing multiprotein complexes [11], and subsequently described in a large number of organisms. Cross-species studies revealed that Alp4/6, the homologues of GCPs 2 and 3 in fission yeast, can be replaced to a limited extent by the human proteins, or by the budding yeast homologues Spc97/98 [12]. This underlines the high degree of functional conservation of GCPs across species. Because the nomenclature for GCPs often varies for different model organisms [3], we will use here the terminology as applied for human GCPs, to facilitate the comprehension of this review article.

**Figure 1.**
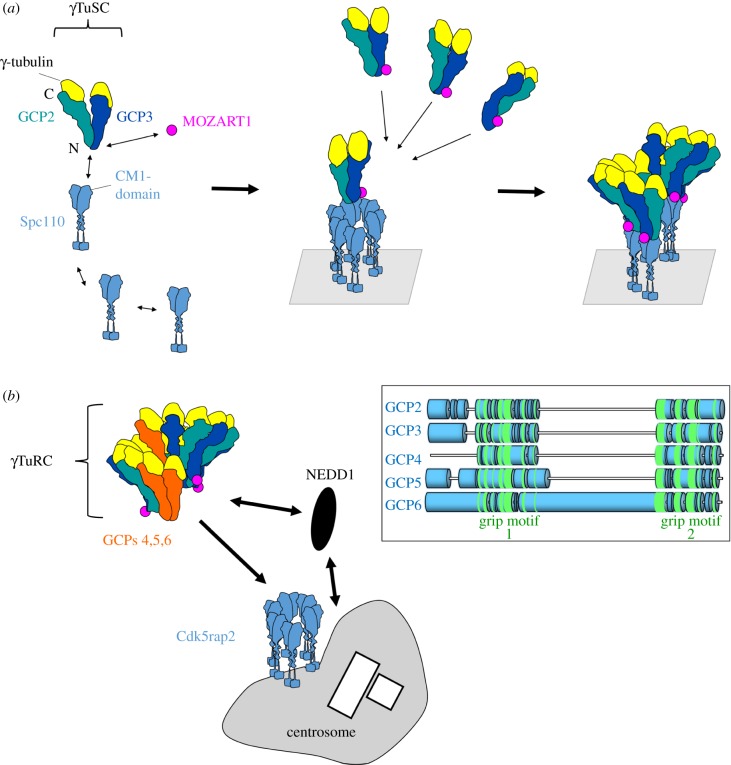
Assembly and recruitment of γ-tubulin complexes. (*a*) GCP2 and GCP3 interact laterally, and bind longitudinally each to one molecule of γ-tubulin, to form the *γ*TuSC. Assembly of helical complexes from *γ*TuSCs is driven by oligomerization of proteins with a CM1 domain, such as Spc110 in *S. cerevisiae*. The CM1 domain binds to the amino-terminal region of GCP3, together with a small oligomerization-promoting protein, MOZART1. (*b*) Soluble *γ*TuRCs are fully assembled in the cytoplasm, and are recruited to the centrosome by NEDD1 and by CM1 proteins, such as Cdk5rap2 in mammals. The inset depicts schematically sequence similarities between GCPs 2, 3, 4, 5 and 6. Conserved secondary structures are found in the amino-terminal grip1 domain, and in the carboxy-terminal grip2 domain (highlighted in green). GCPs 5 and 6 contain unique sequence extensions at their extreme amino-termini and between the grip1 and grip2 domains that are not shared with any other GCPs.

Sequence analysis, crystallography and structure prediction have indicated that GCPs 2, 3, 4, 5 and 6 belong to a family of structurally related proteins [13,14]. These GCPs contain two principal conserved domains, grip1 and grip2, located in the amino-terminal and carboxy-terminal halves of the GCPs, respectively (figure 1) [14]. Each grip domain contains multiple bundles of α-helices, with the grip1 domain involved in lateral contacts between GCPs and the grip2 domain mediating binding to γ-tubulin. Two different types of γ-tubulin complexes exist that are defined by size and protein composition: ‘γ-tubulin small complexes' (*γ*TuSCs, approx. 300 kDa) and ‘γ-tubulin ring complexes' (*γ*TuRCs, approx. 2 MDa) [15]. *γ*TuSCs are hetero-tetramers, composed of laterally associated GCP2 and GCP3, each binding longitudinally one molecule of γ-tubulin (figure 1*a*). *γ*TuRCs consist of several *γ*TuSCs that assemble together with GCPs 4, 5 and 6 into a helical structure resembling a ‘lock washer', with the start and the end of the helix overlapping after a single turn [11,16–19]. *γ*TuRCs thus appear like a ring when viewed from the top by electron microscopy. Whereas *γ*TuSC components have been identified in all eukaryotes, the *γ*TuRC-specific GCPs 4, 5 and 6 are missing in a variety of organisms, such as in budding yeast or *Caenorhabditis elegans*. GCPs 5 and 6 exist in single copies in the *γ*TuRC, whereas two copies of GCP4 may be present [20,21]. GCPs 4 and 5 can bind laterally to each other independently of *γ*TuSCs, and together with GCP6 integrate into the wall of the *γ*TuRC helix (figure 1*b*), where they limit and stabilize the size of the complex [14,22,23]. *γ*TuRCs contain several proteins in addition to GCPs, namely MOZART1, MOZART2a/b, NEDD1/GCP-WD, Cdk5rap2/Cep215 and NME7 [21,24–29]. These components are believed to be more peripheral and to have a regulatory function, taking part in assembly, recruitment or activation of the complex.

*In vitro*, the nucleation activity per mole of *γ*TuRC is 150 times higher than that of a *γ*TuSC [15]. This elevated nucleation is likely favoured by the geometry of the *γ*TuRC, with a helical pitch and a 13-fold symmetry that matches the geometry of most microtubules in cells, containing 13 protofilaments of *α*/β-tubulin arranged in a cylinder with a ‘B-lattice'. For this reason, *γ*TuRCs act as direct templates upon activation, by orienting the association of new *α*/β-tubulin dimers. Single *γ*TuSCs may not nucleate microtubules efficiently unless they form oligomers. Oligomerization of seven *γ*TuSCs into a helix is needed to acquire the geometry of a microtubule template, but this necessitates that other proteins occur efficiently [30,31].

## 3. Assembly of γ-tubulin complexes in fungal organisms

In several fungal organisms, oligomerization of *γ*TuSCs is supported by a class of adaptor proteins at the spindle pole body and at cytoplasmic MTOCs. These proteins include Spc110 and Spc72 in *Saccharomyces cerevisiae* and *Candida albicans*, Pcp1 and Mto1 in *Schizosaccharomyces pombe* and ApsB in *A. nidulans* [32]. They possess a conserved sequence motif of approximately 60 amino acids in their amino-terminal region, termed CM1 (centrosomin motif 1) [33]. This motif interacts with the amino-terminal domain of GCP3 with very high affinity [34,35]. Moreover, a subgroup of CM1 proteins contains a second amino-terminal motif called SPM (Spc110/Pcp1 motif) that cooperates in *γ*TuSC-binding [34]. CM1 proteins form coiled-coil dimers that associate laterally into higher oligomers, to build a scaffold for *γ*TuSC oligomerization (figure 1*a*). In *S. pombe*, the CM1 protein Mto1 requires a second protein, Mto2, for efficient oligomerization [36,37]. To anchor *γ*TuSCs to their respective MTOCs, CM1 proteins carry specific sequence motifs in their carboxy-terminal region [34]. In the final multiprotein complexes with *γ*TuSCs, 13 copies of CM1 proteins are present at an equimolar ratio with γ-tubulin [37]. CM1 proteins can, therefore, be considered as structural templates to direct *γ*TuSC oligomerization into nucleation-competent, helical structures [34,35,37]. MOZART1, a small protein without CM1 sequence, also interacts with the amino-terminal domain of GCP3 and cooperates with CM1 proteins to promote *γ*TuSC oligomerization [38,39]. In models where GCPs 4, 5 and 6 are either absent (*S. cerevisiae* and *C. albicans*) or non-essential (*S. pombe*, *A. nidulans*), *γ*TuSCs are the minimal subunits needed for microtubule nucleation.

## 4. Assembly of γ-tubulin complexes in other eukaryotes

In many eukaryotes, microtubule nucleation requires the presence of pre-assembled *γ*TuRCs, comprising GCPs 4, 5 and 6. Experiments in which the expression of a single one of these GCPs is inhibited lead to the disappearance of *γ*TuRCs in favour of smaller complexes at the size of *γ*TuSCs, as seen by fractionation of the cytoplasm on sucrose gradients [23,40,41]. This suggests that GCPs 4, 5 and 6 are necessary either for the initial assembly of *γ*TuRCs, for their stabilization after assembly, or both. Besides, it has been suggested that the assembly of *γ*TuRCs in human cells also depends on MOZART1 [39], but depletion experiments in different cell lines led to conflicting results [39,41].

Depletion of GCPs 4, 5 or 6 is usually accompanied by decreased recruitment of γ-tubulin complexes to the centrosome and induces defects in centriole duplication and spindle bipolarity [23,41,42]. Nevertheless, in somatic cells of *Drosophila melanogaster* bipolar spindles still form in the absence of GCP 4, 5 and 6, and *γ*TuSC proteins are still recruited to mitotic centrosomes [40,43]. This suggests that local oligomerization of *γ*TuSC into helices may occur at certain MTOCs, as described above in fungi, and that microtubules can be nucleated by alternative pathways, either from *γ*TuRCs or from *γ*TuSCs. In vertebrate cells, however, the *γ*TuRC pathway seems to be prevalent.

Nevertheless, helical complexes in the form of *γ*TuRCs or *γ*TuSC oligomers are not sufficient to initiate microtubule nucleation in the cell, because binding to distinct effector proteins is needed for controlled activation.

## 5. Activation of γ-tubulin complexes

Because *γ*TuRCs are present as full-sized complexes in the soluble fraction of the cytoplasm, the problem arises as to how the cell controls their nucleation activity, because the formation of microtubule networks is considered to be spatially restricted and cell cycle-dependent. The percentage of active γ-tubulin complexes may be as low as 1%, concentrated at MTOCs [44–46]. Thus, activation of *γ*TuRCs should coincide with recruitment to MTOCs. *γ*TuRC recruitment to the centrosome or to non-centrosomal MTOCs such as the Golgi apparatus or mitochondria involves proteins that carry CM1 sequence motifs [26,47–50]. The structure of these proteins is similar to their fungal orthologues, with an amino-terminal CM1 motif and a carboxy-terminal MTOC-targeting motif. Examples include *Drosophila* centrosomin, as well as vertebrate Cdk5rap2, myomegalin and pericentrin [26,33,34,47,48,51].

Part of the activation mechanism of γ-tubulin complexes is thought to involve a conformational change in GCP3. A swivel of the carboxy-terminal half of GCP3 results in the lateral alignment of its carboxy-terminally bound γ-tubulin molecule with neighbouring γ-tubulins, to match the geometry of the microtubule cylinder [52,53]. Likely, this activation is triggered by an allosteric mechanism, for example by binding of the CM1 domain to the amino-terminal region of GCP3. As a proof of concept, forced alignment of the γ-tubulin subunits by chemical cross-linking increases the nucleation activity of the complex [53]. Although the insight into this activation comes from studies of yeast *γ*TuSCs, it is likely that equivalent mechanisms drive activation of *γ*TuRCs in higher eukaryotes, given the high structural resemblance of GCPs between different species. An additional role in the activation of nucleation has been attributed to NME7 kinase activity [29]. Because NME7 associates both with soluble, inactive *γ*TuRCs and with centrosome-bound, active *γ*TuRCs, the question arises as to what triggers NME7 activity upon binding to the centrosome.

It has been shown that the activation of nucleation can be uncoupled experimentally from *γ*TuRC recruitment, by expressing protein fragments containing the CM1 domain of mammalian Cdk5rap2 [21]. Cdk5rap2 binds to the pre-formed *γ*TuRCs, and binding requires the interaction of MOZART1 with the amino-terminal domain of GCPs [21,26,39,41]. The isolated CM1 domain (also called *γ*TuNA for ‘*γ*TuRC nucleation activator') has the potential to induce microtubule nucleation from soluble γ-tubulin complexes *in vitro* or in the cytoplasm. Cdk5rap2 may thus fulfil a dual role: as an adaptor for the anchorage of γ-tubulin complexes to specific MTOCs, and as an activator of the anchored complexes. In this context, a recent study showed that knockdown of Cdk5rap2 in primary keratinocytes weakly affected recruitment of *γ*TuRCs to centrosomes, but drastically reduced microtubule nucleation from there [54]. It was concluded that Cdk5rap2 is mainly responsible for activation but not anchorage of *γ*TuRCs to the centrosome. Anchorage was rather attributed to NEDD1 which is not required for *γ*TuRC assembly, but associates with the pre-formed complex in a MOZART1-dependent manner, similarly to Cdk5rap2 [41]. NEDD1 was shown to be an important recruitment factor in interphase and in mitosis [24,25,54,55]. In primary keratinocytes, knockdown of NEDD1 was recently claimed to reduce centrosomal localization of γ-tubulin without significantly affecting centrosomal microtubule nucleation [54], but this interpretation contradicts earlier studies and fails to explain how centrosomes with low levels of γ-tubulin can maintain regular rates of microtubule nucleation [24,25]. The controversy may be partly explained by the observation that different organisms, different cell types or different cellular conditions require different factors for the recruitment and activation of γ-tubulin complexes: for example, NEDD1 is downregulated during differentiation of keratinocytes [54], and multiple genes encode different CM1 proteins, of which individual ones are expressed under several splice variants in a tissue-specific manner [49,50,56,57]. Moreover, changes in expression levels or post-translational modifications may alter the interaction between *γ*TuRCs and regulatory proteins in the same cell throughout the cell cycle, as described for the ratio of MOZART1 bound to *γ*TuRCs in *S. pombe* [58].

As interactions between *γ*TuRCs, CM1 proteins, MOZART1 and NEDD1 all depend on the amino-terminal domains of GCPs [39,41], there is a possibility of functional redundancy among *γ*TuRC regulators. Thus, individual cell types may compensate altered protein levels of MOZART1 or NEDD1 by the expression of specific CM1 isoforms. Furthermore, individual combinations of regulators may affect the cell's capacity to build nucleation-competent complexes from *γ*TuSCs, or to recruit ready-made *γ*TuRCs. In addition, the presence of these regulators may affect the number and activity of *γ*TuRCs at a given MTOC. As an example, *Drosophila* oocytes and sperm require full *γ*TuRCs, although most other cells in the fly can nucleate microtubules from oligomerized *γ*TuSCs [43]. Another notable example is cells from human patients with TUBGCP4 gene mutations, containing very low protein levels of GCP4 and consequently low cellular amounts of *γ*TuRCs. Patients with these mutations are affected by microcephaly and retinal abnormalities, but without visible defects anywhere else in the body [59]. Similar defects could be reproduced by morpholino treatment against TUBGCP4 in zebrafish [59]. This underlines that reduced amounts of *γ*TuRCs can be tolerated in most cells in the human body, but it remains to be determined whether this is due to a compensation by the remaining *γ*TuRCs, by *γ*TuSC-dependent nucleation, or by alternative nucleation mechanisms independent of γ-tubulin.

## 6. Individual roles of GCPs 4, 5 and 6 at specific microtubule-organizing centres?

Formation of the *γ*TuRC occurs through lateral binding of *γ*TuSCs to the grip1 domains of GCPs 4, 5 and 6 [23]. Thus, GCPs 4, 5 and 6 have structural and functional similarities to the *γ*TuSC components GCP2 and 3 [14]. Nevertheless, GCPs 5 and 6 possess distinct insertions between their grip1 and grip2 domains, and display unique sequence extensions at their extreme amino-terminal ends that differ from GCPs 2 and 3, whereas GCP4 is lacking any additional sequence outside the grip motifs (figure 1*b*) [14]. Because contacts between γ-tubulin complexes and regulatory proteins occur via the amino-terminal regions of GCPs, it is tempting to speculate whether the unique sequence features of GCPs 4, 5 and 6 enable any specific spatio-temporal regulation of *γ*TuRCs that cannot be performed on γ-tubulin complexes composed exclusively of *γ*TuSCs. Consistently, deletion of the genes encoding GCPs 4, 5 or 6 in *S. pombe* specifically weakens the activity of non-spindle pole MTOCs in interphase [22]. Moreover, individual roles for GCP5 and GCP6 have been reported in various experimental systems. GCP6 may act synergistically with MOZART1 for bipolar spindle assembly and faithful chromosome segregation in *S. pombe* [58]. Besides, GCPs 5 and 6 are substrates for multiple kinases that regulate *γ*TuRC-specific functions during the cell cycle [42,60]; for example, GCP6 is phosphorylated by Plk4 at its sequence insertion between the grip1 and grip2 domains, and a non-phosphorylatable mutant specifically impairs centriole duplication, without affecting assembly or centrosomal targeting of the *γ*TuRC [42]. Furthermore, the GCP6-specific sequence insertion has also been implicated in the recruitment of *γ*TuRCs to keratin filaments, to create non-centrosomal MTOCs in epithelial cells [61].

In summary, GCPs 5 and 6, and possibly GCP4, may not only play a structural role in *γ*TuRC assembly but also mediate spatio-temporal regulation of *γ*TuRC activity, by interacting with particular regulators or adaptors, during specific phases of the cell cycle, or in specific cell types.

## 7. Microtubule nucleation from augmin-bound *γ*TuRCs

**Figure 2.**
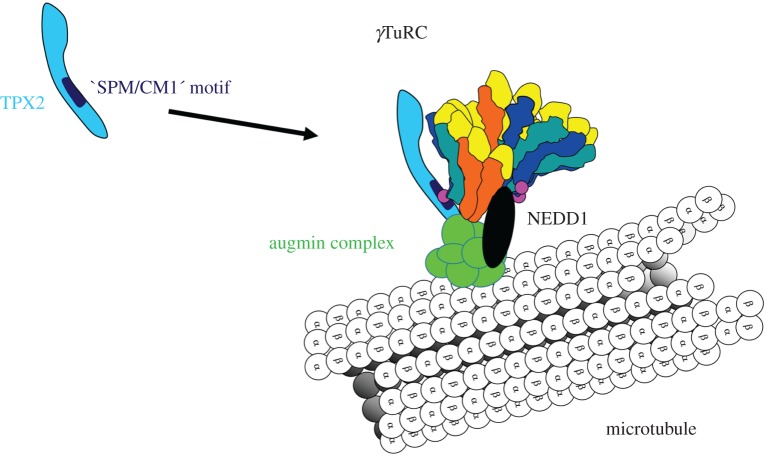
Augmin complexes recruit *γ*TuRCs to the surface of spindle microtubules, to initiate nucleation of ‘secondary microtubules'. Augmin-dependent recruitment occurs in the presence of NEDD1 and the spindle assembly factor TPX2. TPX2 interacts with the *γ*TuRC via a composite binding sequence that bears resemblance to the SPM and CM1 motifs of yeast Spc110.

In many eukaryotes, the presence of full-sized *γ*TuRCs is essential for the nucleation of microtubules from the surface of existing ones. In *Drosophila*, *γ*TuSCs can still be recruited to the centrosome in the absence of GCP4, 5 or 6, but not to the surface of spindle microtubules, where nucleation of ‘secondary microtubules' occurs, to increase the microtubule density of kinetochore fibres [40]. This ‘secondary nucleation' is driven by *γ*TuRCs, laterally attached to the lattice of spindle microtubules (figure 2). The attachment is mediated by augmin multiprotein complexes that are conserved in animals, plants and fungi, but that have been lost during evolution of budding and fission yeast [62,63]. In humans, the augmin complex comprises 8 subunits termed HAUS1–8, and association with the *γ*TuRC involves binding of the HAUS6 subunit to the recruitment factor NEDD1 [64,65]. In addition, augmin has been found to associate with TPX2, a spindle assembly factor that ensures high density of spindle microtubules and the correct formation of spindle poles (figure 2) [66,67]. Interestingly, a region within the TPX2 sequence has been identified that bears similarities to a combination of an SPM motif with a CM1 domain [68]. Different from ‘classic' CM1 proteins such as Spc110, this composite TPX2 motif is located in the carboxy-terminal region of the protein, and the SPM part overlaps with a sequence that resembles the first half of a regular CM1 motif, of which the last six amino acids are further separated by an unstructured stretch of 30 amino acids [68]. As deletion of this composite motif from TPX2 inhibits the branching of secondary microtubules, the mechanism of *γ*TuRC activation by augmin/TPX2 may be comparable to centrosomal activation mechanisms, involving ‘classic' CM1 proteins.

## 8. Influence of microtubule-associated proteins on the nucleation process

**Figure 3.**
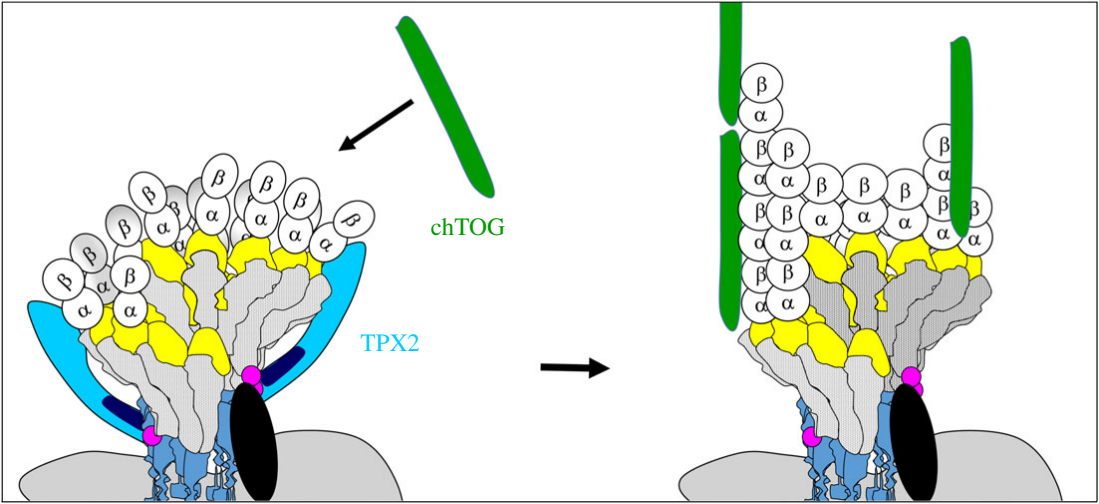
Efficient formation of microtubules from *γ*TuRCs requires additional proteins that interact with early intermediates of nucleation. At early stages of nucleation, single tubulin dimers bind to the *γ*TuRC, some of which are lacking lateral interactions. These early nucleation intermediates are stabilized by TPX2 until a closed tube is formed, independent of the *γ*TuRC-binding property of TPX2. In the next step, tubulin polymerization is supported by the microtubule-associated protein chTOG (= XMAP215 in *Xenopus laevis*).

Compared to spontaneous microtubule nucleation from pure tubulin, the presence of *γ*TuRCs accelerates the nucleation rate significantly [69]. Nevertheless, there remains a lag phase at the beginning of this process, indicating that *γ*TuRCs are not the perfect templates, despite a geometry that resembles the microtubule [70]. Moreover, purified *γ*TuRCs are poor nucleators *in vitro*, even if activity-enhancing conformational changes are induced experimentally [53]. This imperfection may be due to the fact that newly bound *α*/β-tubulin dimers have a slightly curved conformation, bending outwards and lacking lateral contacts with neighbouring dimers. Such nucleation intermediates may be unstable until lateral contacts are formed and the microtubule cylinder closes. This early, unstable phase can be shortened if nucleation intermediates are stabilized by TPX2, by direct binding to tubulin, independent of the presence of *γ*TuRCs (figure 3) [70–72]. The presence of another microtubule-associated protein, chTOG (also known as XMAP215 or Msps in other species), has a synergistic effect in this process, because it supports polymerization (figure 3) [71]. Nucleation of microtubules in cells thus involves multiple steps: (i) the formation of a template that resembles microtubule geometry, in the form of a helical γ-tubulin complex; (ii) the activation of this template, probably by triggering a conformational change in GCP3; and (iii) stabilization of early nucleation intermediates, by favouring specific conformations of tubulin dimers, and by supporting lateral interactions between dimers, to drive closure of the microtubule cylinder.

## Data accessibility

This article has no additional data.
